# Hypertension and Cardiovascular Disease: Is a Treatment Strategy Focused on High Risk Sufficient?

**DOI:** 10.1111/jch.13324

**Published:** 2018-07-01

**Authors:** Daniel W. Jones

**Affiliations:** Sanderson Chair in Obesity, Metabolic Diseases and Nutrition, Director, Clinical and Population Science, Mississippi Center for Obesity Research, Professor of Medicine and Physiology, University of Mississippi Medical Center, 2500 North State Street, Jackson, MS 39216, 601-815-8995

**Keywords:** Hypertension, Cardiovascular Disease, Guidelines

The epidemic of cardiovascular disease continues to explode. Cardiovascular disease now accounts for more than a third of deaths world-wide creating a global health crisis that must be addressed.^1^ The epidemic is driven by lifestyle changes over the last few decades including calorie and sodium dense foods and a decrease in physical activity. Growing trends of overweight and obesity are associated with rapidly rising prevalence rates for hypertension, diabetes, and dyslipidemia.^2^ The obvious best solution for the problem is a reversal of these lifestyle issues leading to primordial prevention of risk factors and cardiovascular disease. However, several decades after recognizing this need and call for this change, the trends are in the wrong direction. Across the globe, more and more countries are facing the growing epidemic of obesity and the associated disorders. Gaining control of the root cause of the epidemic of cardiovascular disease will require a better effort including leadership from the science and medicine community, better efforts at health education and literacy, and strong political and policy actions at the local, national, and global levels. What follows in this commentary can be considered a second best option (a temporizing measure) for addressing the issue while we deal with attempts to improve diet and physical activity.

As the rates of cardiovascular disease and its known risk factors continue to increase globally, in the United States and other countries with advanced economies, age-adjusted CVD death rates have improved over the last 2-3 decades.^3^ Most of the improvement has come through an approach of treating patients with the identified highest risk.^4^ These strategies leading to improved rates have included the use of catheter based interventions such as angioplasty and thrombolytic therapy for acute events including myocardial infarction and stroke as well as better management of hypertension and dyslipidemia. But, in the United States, progress has slowed in recent years. Cardiovascular disease remains the leading cause of death, and CVD mortality rates have begun to plateau. These trends indicate we are likely approaching the limits of progress attainable through existing treatment strategies.

Part of what has driven the strategy of focusing on high risk patients is the reliance on evidence from randomized controlled event based clinical trials (RCTs). Over the last few years, guideline groups across the globe have moved from consensus-based guidelines to evidence-based guidelines. Almost all guideline groups have focused on event-based randomized trials as the best form of evidence. The era of evidence-based medicine has certainly moved us forward in many ways. However, there may be a fly in the ointment when it comes to reliance on this strategy in terms of managing hypertension. For the population of patients at risk from hypertension, the current strategy leaves a large residual risk.

In countries with effective programs of hypertension control, much progress has been made in recent years. This progress is marked by better hypertension control rates and a decrease in the mean blood pressure for the population at large leading to lower age adjusted cardiovascular disease event rates and death rates.^5^ In the US, these changes have been associated with important changes in the relationship between blood pressure and cardiovascular events at the population level. In pooled data from three large observational studies performed in the 1980s and 1990s, most of the CV events occurred in patients with a BP ≥140/90. Pooled data from three studies from the 2000s reveal a majority of CV events occurring in patients with BP < 140/90 (Table 1).^6^

Data from both observational studies and meta-analyses of achieved BP in RCTs demonstrate a continuous relationship between BP and CVD risk beginning at a SBP of about 115 mm Hg.^7^ It has not been feasible to perform event-based RCTs in young patients at lower risk. All the evidence from RCTs about hypertension therapy and CVD risk is from older patients at higher total risk because this is the population easiest to study and demonstrate benefit. A significant portion of the residual risk is related to BP treatment goals that fall short of ideal physiologic BP levels.

The relationship between BP and CVD risk is continuous. It is important to note that in most countries, SBP tracks with age. The increase in SBP with age is seen in all countries where weight increases with age and the diet is sodium dense. It is important to note that this increase in BP with age is neither physiologic nor inevitable.^8^ Because BP tracks with age, relative to other risk factors, BP levels in young adulthood not only predict future BP, mortality risk is predicted as well.^9^ (Figure 1) We also know that both lifestyle therapy and pharmacotherapy in patients with SBP 120-140 can attenuate the rise in BP with time.^10,11^ Though short of evidence from event based trials in young patient, the evidence is compelling that intervening on BP at lower levels and younger ages could attenuate the rise in BP and prevent cardiovascular events in middle and older age.

An important step in dealing with some of the residual risk from hypertension was taken with the 2017 ACC/AHA Blood Pressure Management Guidelines.^12^ The reduction in the treatment goal from a SBP of 140 mm Hg to 130 mm Hg offers opportunities to further reduce risk. However, the 2017 guidelines call for use of pharmacotherapy in patients with a SBP 130-140 mm Hg only in those with a 10-year CVD risk ≥ 10%. Because of the lack of evidence from RCTs for lower risk patients, the guidelines suggest lifestyle therapy only for patients at lower 10-year risk. This is not from evidence that there is no benefit, but from the lack of trials addressing the question.

These realities raise important questions. Will current strategies lead to the reduction in CVD event and death rates that are ideal? Are there opportunities to craft strategies that go beyond the evidence from RCTs, especially since it is unlikely early treatment of patients at lower risk will be tested in the near future? Can we leverage the availability of inexpensive, effective, and relatively safe pharmacotherapy to address the issue of residual risk? And, while we are considering this, can we make more progress on the core lifestyle issues driving the epidemic?

It is unlikely that guideline groups will soon move away from reliance on evidence from randomized controlled trials for offering recommendations for management of hypertension. I am both a co-author and enthusiastic endorser of the 2017 ACC/AHA guidelines. But I do offer suggestions for clinicians to consider that go beyond these recommendations based exclusively on evidence from RCTs. (1) Offer patients maximum opportunities for responding to lifestyle therapy including advice from a dietician. (2) Regardless of calculated 10 year risk, in patients who do not achieve a SBP ≤ 130 mm Hg after 6 months of lifestyle therapy, consider the addition of pharmacotherapy to achieve goal BP. (3) In patients with a family history of premature CVD or hypertension, dyslipidemia, or diabetes mellitus, if a SBP ≤ 120 mm Hg is not achieved with lifestyle therapy, consider the addition of pharmacotherapy. (4) Join the World Hypertension League in their efforts to bring about policy changes leading to a healthier food and physical activity environment in countries around the globe.

Implementation of these strategies could go a long way toward reducing the residual risk of hypertension in individual patients and the population and move us closer to a goal of lower CVD events across the globe.

## Figures and Tables

**Figure 1:**
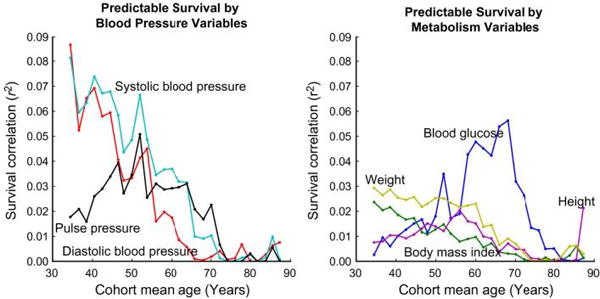
The individual variables’ ability to predict survival changes over time. Blood pressure, BMI, and weight are predictive of mortality primarily from ages 35 to 60 and while blood glucose is most predictive from ages 57 to 73. Predicting all‐cause mortality from basic physiology in the Framingham Heart Study^9^

**Table 1:** Percentage of Incident CVD Events in Persons with Blood Pressure <140/90 mmHg^6^

CVD Event	1980s-1990s	2000s
Stroke	23%	63%
CHD	31%	63%
HF	26%	60%

CVD indicates cardiovascular disease; CHD, coronary heart disease; HF, heart failure.
